# Reproducibility of CT radiomic features in lung neuroendocrine tumours (NETs) patients: analysis in a heterogeneous population

**DOI:** 10.1007/s11547-023-01592-y

**Published:** 2023-01-13

**Authors:** Eleonora Bicci, Diletta Cozzi, Edoardo Cavigli, Ron Ruzga, Elena Bertelli, Ginevra Danti, Silvia Bettarini, Paolo Tortoli, Lorenzo Nicola Mazzoni, Simone Busoni, Vittorio Miele

**Affiliations:** 1 Department of Emergency Radiology, Careggi University Hospital, Largo Brambilla 3, 50134 Florence, Italy; 2 Italian Society of Medical and Interventional Radiology (SIRM), SIRM Foundation, 20122 Milan, Italy; 3 Department of Health Physics, L.Go Brambilla, Careggi University Hospital, 50134 Florence, Italy; 4 Department of Health Physics, AUSL Toscana Centro, Via Ciliegiole 97, 51100 Pistoia, Italy

**Keywords:** Lung carcinoids, Lung cancer, Computed tomography, Radiomics, Ki-67

## Abstract

**Background:**

The aim is to find a correlation between texture features extracted from neuroendocrine (NET) lung cancer subtypes, both Ki-67 index and the presence of lymph-nodal mediastinal metastases detected while using different computer tomography (CT) scanners.

**Methods:**

Sixty patients with a confirmed pulmonary NET histological diagnosis, a known Ki-67 status and metastases, were included. After subdivision of primary lesions in baseline acquisition and venous phase, 107 radiomic features of first and higher orders were extracted. Spearman’s correlation matrix with Ward’s hierarchical clustering was applied to confirm the absence of bias due to the database heterogeneity. Nonparametric tests were conducted to identify statistically significant features in the distinction between patient groups (Ki-67 < 3—Group 1; 3 ≤ Ki-67 ≤ 20—Group 2; and Ki-67 > 20—Group 3, and presence of metastases).

**Results:**

No bias arising from sample heterogeneity was found. Regarding Ki-67 groups statistical tests, seven statistically significant features (*p* value < 0.05) were found in post-contrast enhanced CT; three in baseline acquisitions. In metastasis classes distinction, three features (first-order class) were statistically significant in post-contrast acquisitions and 15 features (second-order class) in baseline acquisitions, including the three features distinguishing between Ki-67 groups in baseline images (MCC, ClusterProminence and Strength).

**Conclusions:**

Some radiomic features can be used as a valid and reproducible tool for predicting Ki-67 class and hence the subtype of lung NET in baseline and post-contrast enhanced CT images. In particular, in baseline examination three features can establish both tumour class and aggressiveness.

## Background

Pulmonary neuroendocrine tumours (NETs) are a group of neoplasms that account for about 25% of all NETs and 2% of lung cancers, they are divided into different groups according to their aggressiveness [1, 2]. In particular, NETs are classified as low-grade or typical carcinoids (TCs), intermediate grade or atypical carcinoids (ACs) and high grade, divided into large-cell neuroendocrine carcinomas (LCNECs) and small-cell lung carcinomas (SCLC) [3]. This division into successively more aggressive forms is based on the progressive increase in the number of mitoses and the presence of necrosis at the histological evaluation: usually typical carcinoid does not show necrosis unlike the atypical ones, whereas the highest percentage of mitoses are found in SCLC, the most aggressive form of NETs [4–7].

Computed tomography (CT) is the imaging of choice in diagnosing this pathology, being able not only to detect changes related to the presence lesion, but also necessary for loco-regional staging of disease [8, 9]. The most frequent findings on CT are the presence of a solid consolidative lesion within the lung parenchyma, frequently polylobate, especially in the case of low-grade forms, with vivid enhancement after administration of contrast medium. The lesion may also present as endo-bronchial or with a mixed parenchymal and bronchial component. Parenchymal atelectasis may also be present in the case of bronchial obstruction [10, 11].

Increasingly important in assessing tumour aggressiveness and thus meaning in the prognosis of these patients by the correlation of nuclear antigen expressed by proliferating cells (Ki-67). Although this classification is not yet part of the grading system of lung NETs, it is currently used in gastrointestinal neuroendocrine tumours (GEP-NENs) according to the 2019 WHO classification. Well-differentiated NENs are further divided into grades based solely on Ki-67 proliferation index and mitotic index: into grade 1 (G1, mitotic rate < 2, Ki-67 index < 3), grade 2 (G2, mitotic rate 2–20, Ki-67 index between 3 and 20) and grade 3 (G3, mitotic rate > 20, Ki-67 index > 20) [12, 13]. It has been studied how these grading values can play a fundamental role in prognostic evaluation and differentiation between various tumour histotypes and in particular in discriminating between TCs and ACs tumours or high-grade SCLS and LCNEC from carcinoid tumours [12, 13].

In recent years, radiomics, with the use of texture analysis, is becoming an increasingly used tool, capable of giving more precise structural information, not always visible by the human eye and not subject to interindividual variability [14–21]. Radiomics is therefore an innovative technique used to characterize the inhomogeneity of a given tissue, and more specifically, as in our case, the lung NET lesions, through the extraction and analysis of features obtained by investigating regions of interest (ROI) from different imaging modalities such as CT, magnetic resonance imaging (MRI) or positron emission tomography CT (PET-CT) [22–26]. The application of texture analysis in NET of the lung could therefore be useful in both the diagnosis and early differentiation of distinctive NET tumour histotypes [27, 28]. In this paper, we faced the reproducibility of CT radiomics features in lung NETs using different CT scanners, trying to integrate different features or to find new metrics in assessing tumour aggressiveness and histotype, that could be reproducible in daily practice.

### Materials and methods

#### Patients and ethics issues

**Fig. 1:**
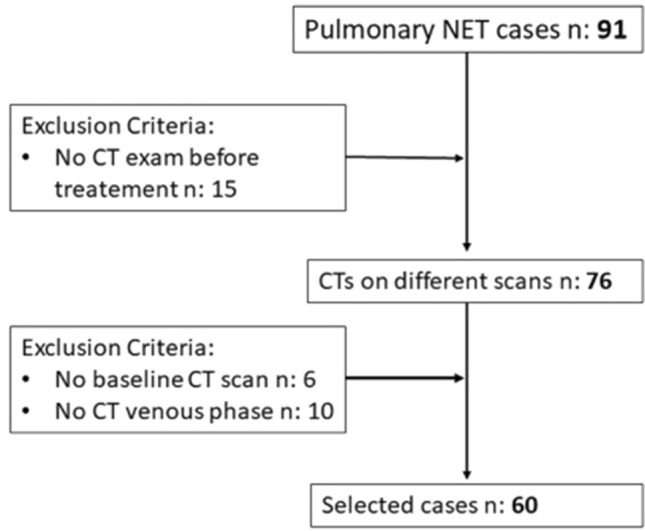
Workflow of patients’ selection

This is a single-centre, observational, retrospective study. Between September 2008 and October 2021, all patients with a histological diagnosis of pulmonary NET who underwent pre-treatment CT exam were selected by searching our Picture Archiving and Communication System (PACS). Inclusion criteria were: patients aged between 18 and 99 years; histological diagnosis of pulmonary NET confirmed by biopsy or by surgical specimen; Ki-67 value; CT examination performed in our department with non-enhanced acquisition and venous phase; at least one CT or PET-TC in the follow-up. The workflow of patient’s selection is shown in Fig. 1. The initial population included 91 patients; of these 15 had no pre-treatment CT performed in our hospital. To make the sample more homogeneous, we excluded those who did not have a baseline-CT (six patients) and those who did not have venous phase acquisition (10 patients). Our study population resulted in 60 patients, 29 males and 31 females. As there is still no standardization in the use and class division of lung NETs using the Ki-67 index, we relied in our study on the most recent WHO 2019 GEP-NENs grading. Selected patients were then divided in three groups: Ki-67 < 3 (Group 1), 3 ≤ Ki-67 ≤ 20 (Group 2) and Ki-67 > 20 (Group 3). This retrospective observational study was approved by the Ethics Committee of our Institution (study protocol n:14776_oss).

#### Images acquisitions and analysis

CT images were acquired using different CT scanners, as shown in Table 1. The study protocol consisted of a baseline acquisition followed by a venous phase acquisition with a 70s’ delay after administration of an intravenous contrast medium (flow 3 mL/s, followed by bolus of saline with a dose of 40 ml). Two types of contrast medium were injected, Ultravist®370 (Bayer Healthcare) and Iomeron®400 (Bracco Imaging Italia). The acquisition parameters for the basal scan were: matrix size 512 × 512 pixels with slice thickness between 1 and 5 mm, 120 kVp, 145 ± 97 mAs, CTDIvol of 10.3 ± 6.7 mGy and DLP in the range 237–1226 mGy*cm; for contrast enhanced scans acquisition parameters were: matrix size 512 × 512 pixels with slice thickness between 1 and 5 mm, 120 kVp, 156 ± 101 mAs, CTDIvol of 11.4 ± 6.6 mGy and DLP in the range 253.5–1228 mGy*cm. All studies were reviewed by two radiologists, with 5 and 15 years’ experience in thoracic imaging. The entire volume of the primary tumour was visually segmented in both unenhanced and enhanced acquisitions employing a volumetric ROI (region of interest) using 3DSlicer software version 4.10.2 (open source software; https://www.slicer.org/). The ROI was delineated slice by slice for each patient. Textural features extraction was carried out by means of SlicerRadiomics tool. A total of 107 features of the PyRadiomics lists were selected, belonging to first-order, 3D shape-based, grey level co-occurrence matrix (GLCM), grey level size zone matrix (GLSZM), grey level run length matrix (GLRLM), neighbouring grey tone difference matrix (NGTDM) and grey level dependence matrix (GLDM) classes.

**Table 1:** List of computed tomography (CT) scanners used in our study

CT scanners
Bright Speed, Optima CT 660, LightSpeed VCT, Revolution HD—*General Electric Healthcare*
SOMATOM Emotion 16, Definition Flash, Definition AS + , Sensation 16, Sensation 64, Sensation Open—*Siemens Healthineers*
iCT SP—*Philips Healthcare*

#### Statistical analysis

The intrinsic heterogeneity of the database due to the use of different contrast agents, scanners and reconstruction kernels could lead to the presence of bias in the statistical differentiation of the patient classification groups; to verify the absence of this bias a Spearman’s correlation matrix with Ward’s hierarchical clustering was created using R software (https://www.R-project.org/). Nonparametric tests were performed to identify features that showed significant differences between the three classes of Ki67 (Ki-67 < 3 (Group 1), 3 ≤ Ki-67 ≤ 20 (Group 2) and Ki-67 > 20 (Group 3)) or between the presence or absence of mediastinal lymph-node metastases. This statistical analysis was performed separately on unenhanced and on contrast enhanced CT scans databases using SPSS (IBM SPSS Statistics for Windows, version 27.0. Armonk, NY: IBM Corp). For the Ki-67 class distinction, the Kruskal–Wallis test was used and the post hoc analysis was performed with the Dunn’s test, considering the Bonferroni correction. For the metastases grouping distinction, the Mann–Whitney test was employed. Significance threshold was set at *p* = 0.05.

## Results

**Fig. 2:**
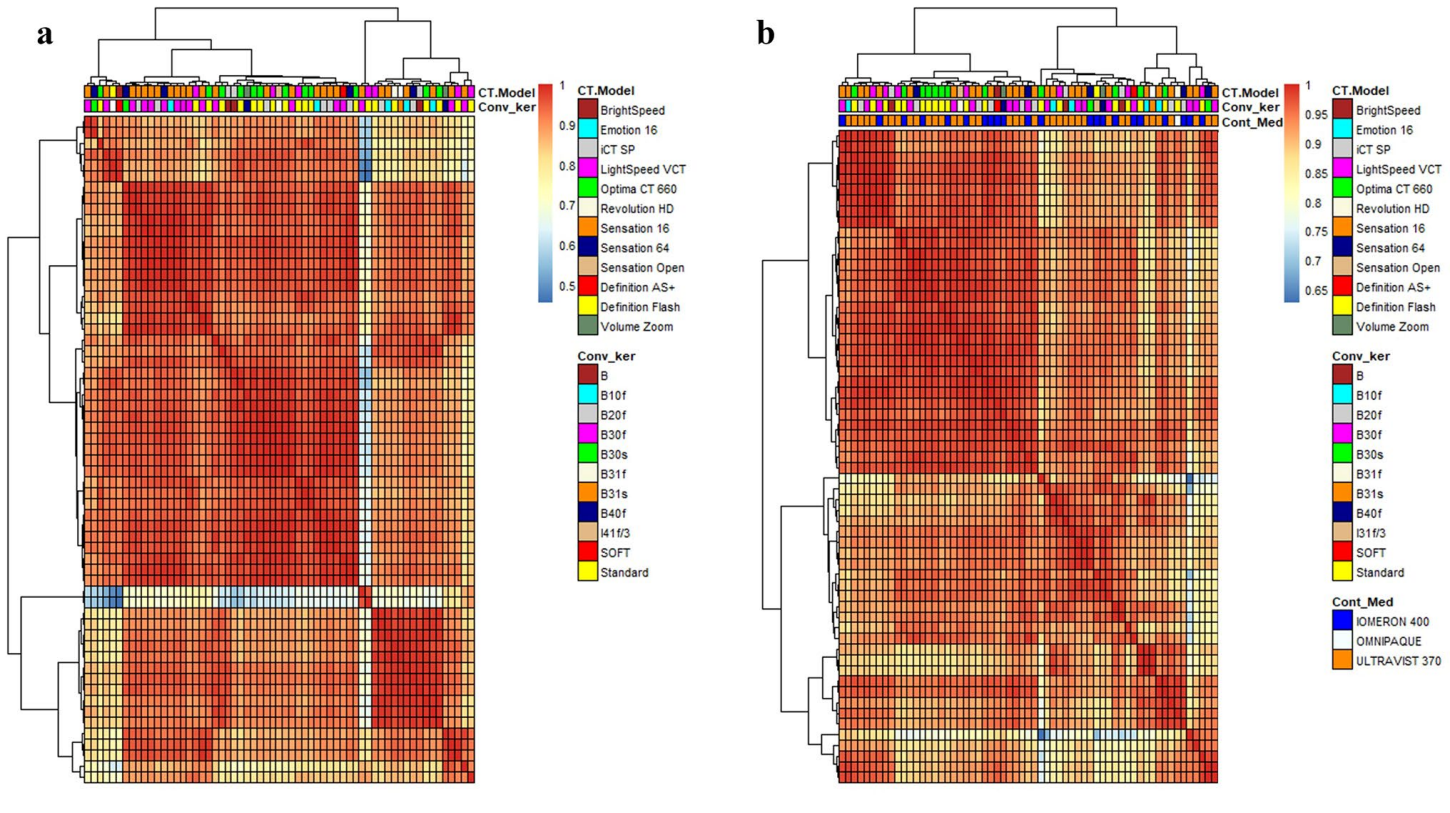
Heatmaps. Heatmap representing Spearman’s correlation matrix with Ward’s hierarchical clustering for unenhanced (**a**) and contrast enhanced (**b**) CT acquisitions. The colour of the heatmap goes from red (high correlation) to blue (low correlation). The top of the matrix shows the dendrogram of the clustering and three coloured bars which represent exam characteristics that could lead to a bias in the statistical correlation between groups (CT model, convolution kernel and contrast medium). It can be seen that all of those characteristics are randomly distributed over the database and none of them can be associated to a specific cluster

This retrospective observational study was approved by the Ethics Committee of our Institution (study protocol n:14776_oss). The 60 patients selected were aged between 35 and 90 years old (mean age 71 years; 35 women, 25 men). Histopathological analysis revealed 45 cases of TCs (75.0%), 12 SCLCs (20.0%) and 3 LCNECs (5.0%). According to Ki-67 expression, patients in Group 1 were 18/60 (30.0%), Group 2 24/60 (40.0%) and Group 3 18/60 (30.0%). Typical carcinoids showed mean Ki-67 values of 7.02, while SCLCs of 74.58 and LCNECs of 80,0. Mediastinal lymph-nodal metastases were present in 30/60 (50.0%) at the first follow-up made by CT or PET-CT. The absence of bias in the database due to possible confounding factors was verified through Spearman’s correlation matrix with Ward’s hierarchical clustering, as shown in Fig. 2. No evidence of a bias associated to these factors was found. In Tables 2 and 3, the features that showed significant differences among the Ki-67 classes, after Kruskal–Wallis with Dunn’s post hoc test, are listed for unenhanced and contrast enhanced CT scans, respectively (Tables 2, 3). The list of significant features, resulting from the Mann–Whitney test, in the distinction between the absence (0) and presence (1) of metastases, is also reported.

**Table 2:** List of features that showed significant differences among the Ki-67 classes in Kruskal–Wallis test for unenhanced CT scans

Feature	Ki67 class	Median	1° quartile	3° Quartile	*p* value	Dunn’s test
*Baseline-CT*						
	1	0.525	0.33	0.6225		
MCC	2	0.44	0.34	0.5825	0.048	/
	3	0.34	0.265	0.43		
	1	182.46	9.1325	1968.1775		
Cluster prominence	2	17.01	3.4025	194.9325	0.046	1–3
	3	3.66	9.01	18.8		
	1	0.67	0.2075	2.24		
Strength	2	0.385	0.1275	0.9875	0.040	1–3
	3	0.16	0.045	0.215		

Feature	Metastasis	Median	1° Quartile	3° Quartile	*p* value	
Grey level variance (gldm)	0	0.83	0.5725	2.04	0.041	
	1	0.58	0.415	0.885		
Small dependence emphasis	0	0.05	0.04	0.0675	0.025	
	1	0.04	0.03	0.05		
MCC	0	0.505	0.345	0.6525	0.016	
	1	0.36	0.285	0.47		
Sum squares	0	0.715	0.5	2.1	0.048	
	1	0.53	0.395	0.845		
Cluster prominence	0	39.575	5.3075	1152.3575	0.033	
	1	9.01	3.69	21.935		
Imc1	0	-0.1	-0.16	-0.07	0.048	
	1	-0.07	-0.12	-0.045		
Cluster tendency	0	1.895	1.1625	6.6975	0.041	
	1	1.16	0.905	2.225		
Variance	0	468.395	310.345	1196.237	0.039	
	1	306.71	195.815	500.74		
Grey level variance (glrlm)	0	0.995	0.7175	3.0175	0.045	
	1	0.71	0.525	1.005		
Grey level non uniformity normalized (glszm)	0	0.205	0.1325	0.27	0.017	
	1	0.26	0.215	0.32		
Grey level non uniformity (glszm)	0	7.8	4.64	21.1475	0.017	
	1	23.82	8.765	40.975		
Large area emphasis	0	7606.69	1944.933	34,346.72	0.047	
	1	40,481.62	5396.74	115,192.56		
Zone percentage	0	0.05	0.03	0.07	0.035	
	1	0.03	0.02	0.05		
Large area low grey level emphasis	0	189.485	92.2725	1300.74	0.035	
	1	1181.95	244.225	2984.82		
Strength	0	0.67	0.225	2.12	0.003	
	1	0.15	0.05	0.415		

For each feature are reported median and Tukey’s 1° and 3° quartile for each class, *p* value and classes pairs that passed Dunn’s test, if present. List of significant features, resulting from Mann–Whitney test, in the distinction between the absence (0) and presence (1) of metastases. For each feature are reported median and Tukey’s 1° and 3° quartile, separately for the two groups, and the *p* value

**Table 3:** List of features that showed significant differences among the Ki-67 classes in the Kruskal–Wallis test between the Ki-67 classes for contrast enhanced CT scans.

Feature	Ki67 class	Median	1° Quartile	3° Quartile	*p* value	Dunn’s test
* BASELINE-CT*						
	1	0.4115	0.3325	0.58875		
Correlation	2	0.4015	0.294	0.52	0.046	1–3
	3	0.293	0.229	0.411		
	1	93	79	116.5		
Median	2	78	59.75	95	0.004	1–3
	3	60	52.5	70		
	1	233	147.5	378.5		
Maximum	2	196	139	267.25	0.005	1–3
	3	134	122	153		
	1	98.965	83.5675	128.56		
Root mean squared	2	81.3765	67.108	102.3775	0.002	1–3
	3	64.473	58.3855	72.0255		
	1	120.5	102	158.75		
90th Percentile	2	104	84.75	124.2	0.001	1–3
	3	86	79.5	93.7		
	1	63.9	47.5	77.75		
10th Percentile	2	53	35	58.35	0.020	1–3
	3	32	29.5	46		
	1	91.549	74.37	116.8468		
Mean	2	76.712	59.16125	89.25025	0.006	1–3
	3	58.515	52.965	69.818		

Feature	Metastasis	Median	1° Quartile	3° Quartile	*p* value	
Median	0	86.5	66.25	104.75	0.049	
	1	68	55	84.5		
Root mean squared	0	95.6215	72.4565	118.7953	0.045	
	1	71.166	61.829	91.037		
90th Percentile	0	115	94.5	137.4	0.030	
	1	93	83.2	111.5		

For each feature are reported median and Tukey’s 1° and 3° quartile for each class, *p* value and classes pairs that had passed Dunn’s test, if present. List of significant features, resulting from the Mann–Whitney test, in the distinction between the absence (0) and presence (1) of metastases. For each feature are reported median and Tukey’s 1° and 3° quartile separately for the two groups and the *p* value

In the correlation analysis between the different types of tumour and Ki-67 classes, three features were statistically significant in non-contrast enhanced scans: MCC (*p* = 0.048), ClusterProminence (*p* = 0.046) and Strength (*p* = 0.040). While in the contrast enhanced scans, seven features resulted statistically significant: Correlation (*p* = 0.046), Median (*p* = 0.004), Maximum (*p* = 0.005), RootMeanSquared (*p* = 0.002), 90^th^ Percentile (*p* = 0.001), 10^th^ Percentile (*p* = 0.020), Mean (*p* = 0.006). All of them, except for Correlation, are first-order class features.

When assessing the correlation between tumour histotype and the presence of metastases, three characters (Median (*p* = 0.049), RootMeanSquared (*p* = 0.045) and 90^th^ Percentile (*p* = 0.030) were found in the feature’s statistical analysis of the venous phase contrast medium images. In the analysis performed on the unenhanced images, 15 features were statistically significant, including those that distinguished the presence or absence of metastases also in post-contrast medium acquisitions, namely MCC (*p* = 0.016), ClusterProminence (*p* = 0.033) and Strength (*p* = 0.003).

## Discussion

To the best of our knowledge, this is the first study evaluating the efficacy of radiomics in considering the aggressiveness and tumour histotype of pulmonary NETs by analysing the possibility of results’ standardization and reproducibility on different CT scanners, therefore avoiding an important bias due to the heterogeneity of the machine used for imaging. In our previous study, we have selected significant features in Ki-67 classes and aggressiveness stratification, using a small number of patients trying to have the most homogeneous group of subjects, using only one CT scanner [27]. We therefore attempted with this additional study to evaluate a larger number of patients, assessing whether with hierarchical clustering analysis there was any bias resulting from the use of different CTs. Different studies have been conducted to assess how variation in the acquisition parameters and reconstruction techniques of different CTs could affect radiomics features [29]. The study by Meyer et al. assessed how most features can be altered secondary to variations in acquisition parameters: in particular slice thickness showed the greatest impact on the reproducibility of these features. This demonstrates how the selection of reproducible features that are not affected by variations secondary to different technical acquisition parameters is therefore a fundamental factor [30]. One of the main results of our study is that none of the features extracted and selected is affected by bias arising from sample heterogeneity.

An important goal of our study was the identification of features capable of differentiating tumour histotypes according to the Ki-67 value. In particular, three features (*maximal correlation coefficient—MCC*, *ClusterProminence* and *Strength*) were significant in non-contrast acquisitions, as explained in the Results section. *Cluster Prominence* and *MCC* are second-order features belonging to the class of the grey level co-occurrence matrix (GLCM). The former is a measure of the skewness and asymmetry of the GLCM. The latter represents a quantification of the complexity of the texture. *Strength* belongs to neighbouring grey tone difference matrix (NGTDM), representing a measure of the primitives in an image. Equally, these features were also significant in acquisitions without contrast medium in correlating with the presence of metastases.

Considering that the majority of patients have lesions with high contrast enhancement, which is a typical characteristic of all neuroendocrine tumours, an evaluation after the administration of contrast medium is mandatory [31–33]. Concerning the correlation between Ki-67 classes and tumour histotypes in enhanced CT images, six first-order features and one second-order feature were significant. *Median* is the median of grey level intensity within the region of interest and was higher in the class with lower Ki-67 values in relation to the greater presence of solid tissue without necrotic or necrotic areas [34]. *Maximum* represents the highest grey level intensity within the region of interest and even this was higher in typical carcinoids due to the absence of necrosis and greater uptake of contrast medium in a more homogeneous tissue structure. *10*^*th*^ and *90*^*th*^* Percentiles* are mirror of the 10^th^ and 90^th^ Percentiles of the grey level intensity within the region of interest, while *Mean* represents the average grey level intensity within the region of interest. As mentioned above, also these features showed higher values in class 1, therefore representing low-grade tumours. *Root mean squared* (*RMS*) is the square root of the mean of all the squared intensity values. *Median*, *RMS* and *90*^*th*^* Percentile* were also significant when analysing the correlation with the presence or absence of metastases in enhanced images, showing higher values in tumours with no mediastinal lymph-node metastases, underlining their ability to detect low-grade tumours. This represents another important point in our study as it identifies these three features as highly useful in differentiating tumours with a high risk of metastasis and high Ki-67 values: these tumours are therefore more inhomogeneous due to the presence of necrosis or colliquation from those with a low tendency to metastasise and with low grading, thus allowing the early identification of those tumours at high risk [35]. *Correlation* is a second-order feature representing the linear dependency of grey level values to their respective voxels in the grey level co-occurrence matrix (GLCM). Higher values of this feature were found to be present in Group 1: this result needs further investigation, maybe not be related to a real tissue inhomogeneity, not common in typical carcinoids. Other features correlate with the presence of metastases, including *Grey Level Variance* belonging to grey level dependence matrix (*GLDM*), representing the variance in grey level in the image and *Grey Level Variance* of grey level size zone matrix (*GLSZM*) that is the variance in grey level intensities for the zone. Both these features showed higher values in tumours with metastasis in relation to the tissue heterogeneity of poorly differentiated NETs. As evidence of this, *Grey Level non-uniformity* (GLSMZ) also showed higher values in tumours that had metastases and were therefore more aggressive.

This study still has some limits: first of all, we acknowledge the relatively small number of patients and we are collecting further exams for future radiomics applications. Moreover, we lack validation of our results with a control group. Another limit is the segmentation performed manually by one radiologist: even if an expert one, it could be interesting to repeat in the next future these evaluations applying an automatic approach based on deep learning. Finally, enhanced exams were performed using two different types of contrast media that may have affected the CT texture of NETs imaging. However, a previous study by Botta et al. demonstrates that radiomic features were not influenced by different contrast media, in fact this was not investigated in our study [36–38].

In conclusion, texture analysis can be a useful tool in the stratification of lung NET tumour histotypes in correlation with Ki-67 values and the presence of metastases. Limitations resulting from sample inhomogeneities can be overcome by selecting features unaffected by acquisition parameters, making the results reproducible and standardized. Although radiomics is not yet used in clinical practice, it may become in the future a valuable aid in the evaluation of both tumour class and aggressiveness of NETs tumour and, therefore, in decision-making process.

## Data Availability

The datasets used and/or analysed during the current study are available from the corresponding author on reasonable request.

## Abbreviations

NET: Neuroendocrine tumour
CT: Computed tomography
TC: Typical carcinoid
AC: Atypical carcinoid
LCNEC: Large-cell neuroendocrine carcinoma
SCLS: Small-cell lung carcinoma
GEP-NEN: Gastrointestinal neuroendocrine tumour
ROI: Regions of interest
MRI: Magnetic resonance imaging
PET-CT: Positron emission tomography computed tomography
PACS: Picture archiving and communication system
GLCM: Grey level co-occurrence matrix
GLSZM: Grey level size zone matrix
GLRLM: Grey level run length matrix
NGTDM: Neighbouring grey tone difference matrix
GLDM: Grey level dependence matrix
MCC: Maximal correlation coefficient
